# The Associations of Child’s Clinical Conditions and Behavioral Problems with Parenting Stress among Families of Preschool-Aged Children: 2018–2019 National Survey of Child Health

**DOI:** 10.3390/children9020241

**Published:** 2022-02-11

**Authors:** Soyang Kwon, Meghan E. O’Neill, Carolyn C. Foster

**Affiliations:** Ann & Robert H. Lurie Children’s Hospital of Chicago, Chicago, IL 60611, USA; meoneill@luriechildrens.org (M.E.O.); ccfoster@luriechildrens.org (C.C.F.)

**Keywords:** early childhood, externalizing behavior problems, special health care needs, mental, emotional developmental, or behavioral problems, parenting aggravation, NSCH

## Abstract

To understand parental stress resulting from parenting young children, the current literature has primarily focused on families of children with clinical conditions, but has placed far less attention on the general population. The aim of this study was to examine parenting stress related to children’s clinical conditions and behavioral problems in a nationally representative sample of US children aged 3 to 5 years. The study sample included 8454 children aged 3 to 5 years and their parents who participated in the 2018–2019 US National Survey of Child Health (NSCH). Using online/paper NSCH questionnaires, parents reported their children’s special health care needs (SHCN), clinically diagnosed mental, emotional, developmental, and behavioral (MEDB) problems (e.g., anxiety problem, developmental delay), and externalizing behaviors. Parents also reported the frequency of feeling aggravated from parenting the participating child as an indicator of elevated parenting stress. In the sample, the prevalence of elevated parenting stress was 5.1% overall (95% CI = 4.2, 6.0); however, it was significantly higher among parents of children with SHCN (20.8%; 95% CI = 16.7, 24.9), with MEDB problems (24.8%; 95% CI = 19.9, 29.8), and with externalizing behavior problems (14.7%; 95% CI = 11.8, 17.6). A multivariable logistic regression model showed that elevated parenting stress was associated with the child’s SHCN (adjusted odds ratio [AOR] = 2.3; 1.3, 3.9), MEDB problems (AOR = 4.8; 95% CI = 2.5, 9.1), and externalizing behavior problems (AOR = 5.4; 95% CI = 3.1, 9.4). Even in children without SHCN or MEDB problems, externalizing behavior problems were associated with elevated parenting stress (AOR = 6.4; 95% CI = 3.3, 12.7). The findings call for greater attention to subclinical or yet to be diagnosed externalizing behavior problems among the general preschool-aged child population and their underestimated impact on parenting stress.

## 1. Introduction

Parenting is a rewarding but demanding job [1]. The practical and emotional demands of child rearing and caregiving can be stressful [1], particularly when a child exhibits challenging behaviors, such as defiance and avoidance [2]. Externalizing behavior problems tend to decline over time and could be the normative development of a child [3]. However, some behavioral problems persist throughout childhood [4]. In attempts to understand the relationship between parenting stress and child behavioral problems, the literature has primarily focused on parenting stress as a risk factor for a child’s internalizing and externalizing behavior problems [5,6]. However, based on a transactional model of child development [7,8], the bi-directionality of parenting stress and child behavior problems has been recognized [9,10], shedding light on the potential for elevated parenting stress as a consequence of the overwhelming demands for child rearing [2]. Yet, fewer studies have focused on understanding which child-related factors elevate parenting stress [11]. A review by Yorke et al. [12] showed that among parents with children aged 3 years or older with Autism Spectrum Disorder (ASD), parenting stress is high when a child displays behavioral problems. In a nationally representative sample of United States (US) children under the age of 17 years, a substantially higher level of parenting stress was reported among parents of children with ASD or with special health care needs (SHCN) [13,14]. As such, most reported data for parenting stress are from families of children with severe health conditions, such as ASD and SHCN, while limited data are available to understand parenting stress associated with challenging behaviors in children who have other clinically diagnosed mental, emotional, developmental, or behavioral (MEDB) problems (e.g., anxiety problem, developmental delay, conduct problem) or in the general child population without clinical conditions. 

Parenting stress related to a child’s behavioral problems seems elevated more among parents of younger (i.e., preschool-aged) children, compared to parents of older children [10,11]. This is not only because preschool-aged children typically spend more time in the parent’s care than older children [15], but also because parenting stress tends to decrease as parents are better able to manage their parenting stress in combination with a decrease in their child’s behavioral problems over time [2]. Among preschool-aged children, externalizing behavior is particularly important as a behavioral marker of problems with a child’s emotional regulation and social competence [16]. Understanding parental stress from parenting young children is significant, because elevated parenting stress is associated with decreased well-being and increased depressive symptoms among parents [13,17], and is also linked to poor parenting practices [18], which, in turn, can influence adverse child health outcomes, such as adjustment problems and delayed cognitive development throughout childhood [19,20]. For example, behavioral problems have been shown to be associated with an increased risk of child maltreatment, abuse, and neglect among young children with SHCN [21]. 

In the general preschool-aged child population without clinical conditions, subclinical or yet to be diagnosed externalizing behavior problems could affect parenting stress. Previous studies have attempted to elucidate parenting stress associated with externalizing behaviors in preschool-aged children with or without clinically diagnosed conditions. For example, McDaniel et al. [16] reported that child externalizing behavior problems predicted the mother’s elevated parenting stress in a community sample of 183 children aged 1 to 5 years. However, national data are lacking to evaluate the significance of parenting stress associated with a child’s externalizing behavior problems at a national level. To address the gap, the first aim of this study was to examine parental stress from parenting children with and without clinical conditions (SHCN and MEDB problems) in a nationally representative sample of US preschool-aged children. The second aim was to examine whether child externalizing behavior problems were associated with parenting stress, regardless of the presence of the child’s clinical conditions. 

## 2. Methods

### Study Sample

The study sample included children aged 3 to 5 years and their parents who participated in the 2018 and 2019 US National Survey of Child Health (NSCH). The NSCH is a web/mail-based national survey to collect information about multiple aspects of children’s lives, such as child health, health care, and family, in a representative sample of non-institutionalized US children aged 0 to 17 years. The 2018 NSCH was conducted from June 2018 to January 2019. The 2019 NSCH was conducted from June 2019 to January 2020. Randomly selected households across the US were mailed an invitation to complete a short online/paper Screener Questionnaire. The questionnaire asked a household member to identify all children aged 0–17 years living in the household. Only one child per household was randomly selected to be the participant of a Topical Questionnaire. The Topical Questionnaire was completed by an adult who was familiar with the participating child’s health and health care (proxy-respondent). The NSCH procedures and modifications were approved by the US National Center for Health Statistics Research Ethics Review Board and the NORC Institutional Review Board.

The 2018 and 2019 NSCH included 9323 children aged 3–5 years. To focus on parenting stress experienced by parents and to rule out the confounding effect due to the known different associations between a child’s MEDB problems and parenting stress experienced by grandparents [22], we excluded 836 children whose proxy-respondents were not parents (e.g., grandparents, relatives). We further excluded 33 children whose parents did not respond to parenting stress questions. Finally, we included 8454 children (90.7%) for data analysis. 

## 3. Measurements

### 3.1. Special Health Care Needs

Children with special health care needs (CSHCN) is defined as children who have “chronic physical, developmental, behavioral, or emotional conditions, and who also require health and related services of a type or amount beyond that required by children generally” [23]. The CSHCN Screener© was administered to evaluate the qualification of SHCN. The screener was designed to ask parents about a child’s experience of five different health consequences: (1) use or need of prescription medication; (2) above average use or need of medical, mental health or educational services; (3) functional limitations compared with others of the same age; (4) use or need of specialized therapies, such as physical, occupational or speech therapy; and (5) treatment or counseling for emotional, developmental, or behavioral problems (EDB). To qualify as having SHCN, children had to be experiencing one of the five consequences, the consequence had to be due to a medical or other health conditions, and the (expected) duration of the condition had to be 12 months or longer. For example, meeting the EDB criteria was determined using the following questions: “Does this child have any kind of emotional, developmental, or behavioral problem for which he or she needs treatment or counseling?” and “If yes, has his or her emotional, developmental, or behavioral problem lasted or is it expected to last 12 months or longer?” 

### 3.2. Mental, Emotional, Developmental, or Behavioral Problems

Children with MEDB problems [24,25] were identified based on the presence of any of the following 10 conditions: (1) Tourette Syndrome, (2) anxiety problems, (3) depression, (4) behavioral and conduct problems, (5) developmental delay, (6) intellectual disability, (7) speech or other language disorder, (8) learning disability (also known as mental retardation), (9) ASD, and (10) Attention Deficit Disorder or Attention-Deficit/Hyperactivity Disorder (ADD or ADHD). Parents were asked about the presence of each of the 10 conditions using the following questions: “Has a doctor or other health care provider ever told you that this child has the condition?” and “If yes, does this child currently have the condition?” Children with MEDB problems were also identified using the CSHCN Screener© EDB criteria. Together, we considered a child as having a MEDB problem if the child’s parent reported that the child currently had any of the 10 conditions or qualified on the CSHCN Screener© EDB criteria. 

### 3.3. Externalizing Behaviors

Externalizing behavior problems refer to a group of behavior problems that are manifested in a child’s outward behavior and reflect child negatively acting on the external environment [26]. Externalizing behaviors consist of hyperactive, aggressive, and disruptive behaviors [27]. In validated behavior assessment tools for young children, such as the Child Behavior Checklist (CBCL) for ages 1.5–5 years [28] and the Strengths and Difficulties Questionnaire (SDQ) for 2–4 year-olds [29], externalizing behaviors are often evaluated in two subdomains: hyperactivity (attention problems) and aggressive behaviors (conduct problems). For the present study, externalizing behaviors were evaluated using three hyperactivity questions and two aggression questions [30], with five response options: never, sometimes, about half the time, most of the time, and always (scored as 0, 1, 2, 3, and 4, respectively). The three hyperactivity questions were (Externalizing Behavior Question 1 (EBQ1)) “when excited or all wound up, how often can this child calm down quickly?” (reverse-scored), (EBQ2) “compared to other children his or her age, how often is this child able to sit still?” (reverse-scored), and (EBQ3) “how often is this child easily distracted?” The two aggression questions were (EBQ4) “how often does this child lose control of his or her temper when things do not go his or her way?” and (EBQ5) “how often does this child become angry or anxious when going from one activity to another?” Each of the item responses were dichotomized into ≥3 (most of the time) and ≤2 (half the time). Children with a score ≥3 to any of these five questions were considered as having externalizing behavior problems. The number of responses with scores ≥ 3 was also calculated. 

### 3.4. Parenting Stress

Parenting stress was evaluated using the Aggravation in Parenting Scale (APS). The APS was derived from the Parenting Stress Index and the Childrearing Scale [31] to assess the level of frustration and stress that parents experience specifically related to caring for the participating child. The APS has been shown to be reliable (Cronbach α = 0.63) [32] and correlates in expected directions with sociodemographic factors [13]. The following three APS questions were asked to parents: “during the past month, how often have you felt (1) this child is much harder to care for than most children his or her age, (2) this child does things that really bother you a lot, and (3) angry with this child?” For each of the three questions, five response options were given: never, rarely, sometimes, usually, or always (scored as 0, 1, 2, 3, and 4, respectively). Parents with a score ≥3 (usually) to any of the three questions were considered as having elevated parenting stress (parenting aggravation) [24]. 

### 3.5. Other Measures

Prior studies [32,33] have suggested that elevated parenting stress is affected by the demographic characteristics of both the child and the parent, family structure, family economic status, parental education, parental immigration status, and the availability of social support. Therefore, we considered the following as potential covariates: child’s age, child’s race/ethnicity, family structure (two parents currently married, two parents not currently married, or single parent), family income, child’s health insurance status, child’s health insurance type, parent’s age, parent’s sex, parent’s education, parent’s birthplace (US or non-US), and the availability of the parent’s emotional support [32]. Child’s race/ethnicity was categorized into non-Hispanic white, Hispanic, non-Hispanic black, Asian, and other. A federal poverty level (FPL) was calculated based on family income and the number of family members. The FPL variable had substantial missing values: 15.3% in 2018 and 17.6% in 2019. The missing values were imputed using sequential regression imputation methods by the Census Bureau [34]. The FPL variable was categorized into <100% (below poverty), 100 to <200%, 200 to <400%, and ≥400% [34]. Child’s health insurance status was dichotomized into having and not having consistent health insurance coverage during the past 12 months. Child’s health insurance type was categorized into public (including public plus private), private, and uninsured. Parent’s age was categorized into three groups: the lowest quartile (20 to 31 years), two middle quartiles (32 to 40 years), and the highest quartile (≥41 years). Parent’s education level was categorized into ≤high school graduation and >high school graduation. To evaluate the availability of emotional support for parenting, parents were asked whether there was someone that they could turn to for day-to-day emotional support with parenting or raising children during the past 12 months. Parents who responded “yes” to this question were considered as having emotional support for parenting. 

## 4. Statistical Analysis

All analyses were conducted accounting for the complex sample design, such as strata, cluster, and weight, using SAS 9.4 survey procedures (Cary, NC, USA). Descriptive analyses, including frequency analyses, were conducted. Chi-square tests were performed to compare the children’s clinical conditions, children’s externalizing behavior problems, and potential covariates by parenting stress status. A multivariable logistic regression model was built to predict the probability of elevated parenting stress by the two clinical conditions: SHCN and MEDB problems. To examine whether elevated parenting stress would be further explained by a child’s externalizing behavior problems, we fit an additional multivariable regression model, adding the externalizing behavior problem variable as a predictor. We also conducted multivariable logistic regression analyses stratified by status of clinical conditions (with vs. without clinical conditions) and by parent sex (mothers and fathers). Of the potential covariates considered, we selected covariates that were found to be statistically (*p* < 0.05) associated with elevated parenting stress in chi-square tests. Missing data were treated using a listwise deletion method. Odds ratios (ORs) and 95% confidence intervals (CIs) were derived from logistic regression models. 

## 5. Results

The prevalence of high parenting stress was 5.1% (95% CI = 4.2, 6.0): 4.1% responded “always” or “usually” to the question of “this child is much harder to care for than most children his or her age,” 2.0% to the question of “this child does things that really bother you a lot,” and 0.6% to the question of “angry with this child.” As shown in Table 1, participant characteristics between those with and without elevated parenting stress were not statistically different, except that male child sex and living below the poverty level reported significantly higher elevated parenting stress. 

In the study sample, 1227 child participants (13.2%; 9% CI = 11.9, 14.6) were identified as CSHCN and 1020 (12.1%; 95% CI = 10.7, 13.5) were identified as having MEDB problems. The most prevalent MEDB condition among preschool-aged children was a speech or other language disorder (8.9%), followed by developmental delay (5.5%; Supplementary Table S1). Of the 1020 children with MEDB problems, 995 children were identified using the responses for the 10 individual MEDB conditions and 25 additional children were identified using the CSHCN screener© MEB criteria. The proportion of children with externalizing behavior problems (32.6%; 95% CI = 30.5, 34.7) was significantly higher than the proportion of children with SHCN or MEDB problems. Almost half of the children with SHCN or MEDB problems (46.9%) had externalizing behavior problems, while this was the case in 28.0% of children without SHCN and MEDB problems. 

The prevalence of elevated parenting stress was 8 times higher among parents of children with SHCN (20.8%; 95% CI = 16.7, 24.9), 10 times higher among parents of children with a MEDB problem (24.8%; 95% CI = 19.9, 29.8), and 9 times higher among parents of children with externalizing behavior problems (14.7%; 95% CI = 11.8, 17.6), compared to their counterparts without the condition (Table 2). Among families with children without externalizing behavior problems, only 1.4% of parents reported elevated parenting stress. However, as the number of externalizing behavior problems increased, the proportion of parents with elevated parenting stress increased (Supplementary Table S2). 

A multivariable logistic regression model (Model 1 in Table 3) showed that the odds of having elevated parenting stress was 3.1 (95% CI = 2.0, 5.0) for SHCN and 6.7 (95% CI = 4.0, 11.0) for MEDB problems. When the child externalizing behavior problem variable was added into the model (Model 2 in Table 3), the adjusted ORs (AORs) for SHCN and MEDP problems remained statistically significant, although they slightly decreased. Parents of children with an externalizing behavior problem were more likely to have elevated parenting stress (AOR = 5.4; 95% CI = 3.1, 9.4). In the stratified analyses of children with and without clinical conditions, the AOR of elevated parenting stress for child externalizing behavior problems was 4.2 (95% CI = 1.9, 9.3) among those with clinical conditions (Model 3 in Table 3) and 6.4 (95% CI = 3.3, 12.7) among those without clinical conditions (Model 4 in Table 3). The stratified analyses of mother and father respondents consistently showed that SHCN, MEDB problems, and externalizing behavior problems were associated with elevated parenting stress (Supplementary Table S3). 

## 6. Discussion

In a representative sample of US children aged 3 to 5 years, this study found that elevated parenting stress was more common among parents of children with SHCN or MEDB problems, compared to parents of children without such clinical conditions. Externalizing behavior problems were prevalent, not only among children with SHCN or MEDB problems (46.9%), but also among children without SHCN or MEDB problems (28.0%). Regardless of the presence of children’s SHCN or MEDB problems, parents of children with externalizing behavior problems were more likely to have elevated parenting stress. 

This is one of the few national studies to examine parental stress from parenting preschool-aged children. We estimated the prevalence of elevated parenting stress at 5.1% in this representative sample of US families of preschool-aged children. In doing so, we used a newly suggested definition of elevated parenting stress [24], which differs from the definitions used in prior NSCH reports (defined as a response score ≥ 2 on average [32], or ≥2 on all three items, or on average [33]). As a result, the prevalence of elevated parenting stress would not be comparable across the studies. However, consistent with previous findings [35], this study found higher (eight times) prevalence of elevated parenting stress among parents of children with SHCN, compared to parents of children without SHCN. By examining a broader range of clinical conditions that are common among preschool-aged children, this study extends on previous findings [13], reporting a 10 times higher prevalence of elevated parenting stress among parents of children with MEDB conditions, compared to parents of children without MEDB conditions. Parenting stress may be higher in this population for several reasons. MEDB problems are chronic conditions for which treatment, typically child and/or family therapy, is often difficult to obtain [36] and often requires parents to adjust their own feelings, attitudes, and behaviors. Depending on the parent’s own positive or negative coping mechanisms, they may have more or less emotional resiliency during periods of a child’s health crisis or relapses, thereby impacting the parent’s attitudes toward care for the child. These challenges may be heightened due to the well documented additional physical, emotional, and financial stressors known to be experienced by parents of children with SHCN resulting from insufficient health care related supports for their children’s conditions [37,38,39]. 

Externalizing behavior problems are known to peak at the age of 2 years and decrease by school entry [40]. The consequences of untreated externalizing behavior problems during early childhood are significant. In the short term, behavioral problems can interfere with children’s ability to participate successfully when they enter school, placing them at risk for poorer academic outcomes [41]. In the long term, behavioral problems could affect physical and mental health outcomes in later life [42,43]. The present study found that, in a representative sample of US preschool-aged children, approximately 3 in 10 children had externalizing behavior problems. Although externalizing behavior problems were more common (46.9%) among preschool-aged children with SHCN or MEDB problems, they were still common (28.0%) among those without such conditions. Our findings suggest that externalizing behavior problems are common difficulties that many US families encounter and yet, the problems could be clinically under-diagnosed and unrecognized. Screening for externalizing behaviors in primary care settings using a validated and feasible tool for pediatric care use (e.g., the Preschool Pediatric Symptom Checklist [44]) may help parents and clinicians better recognize externalizing behavior problems and intervene with appropriate management strategies. Tiered screening for externalizing behavior problems among children with SHCN and MEDB problems is also warranted. Together, these efforts will improve the identification of these developmentally relevant concerns, enhance provider anticipatory guidance, and encourage parent involvement in training-based programs and other relevant therapeutic options. 

Despite prevalent externalizing behavior problems among preschool-aged children, parental stress from parenting preschool-aged children has received less attention [16]. This is one of the first studies to examine an association between a child’s externalizing behaviors and parenting stress in a general population of preschool-aged children. Previous studies have shown that in children with clinically diagnosed conditions, parenting stress is partly or primarily explained by the child’s behavior problems. Baker et al. [45] prospectively examined parenting stress during the preschool years among 205 children with and without developmental delay and found that parenting stress increased with the extent of the child’s behavioral problems, rather than the child’s developmental delay. In families of children with ASD, some studies showed that the child’s behavior problems were most strongly related to parenting stress, rather than other symptoms or the severity of ASD or adaptive skills [11,46], while other studies showed that both the child’s ASD status and level of externalizing behavior problems were associated with elevated parenting stress [30,47]. Our study findings support that both clinical conditions and externalizing behavior problems are associated with elevated parenting stress in families of preschool-aged children. Further, our study adds that even among families of children without clinical conditions, child externalizing behavior problems are associated with elevated parenting stress, calling for greater attention to subclinical or yet to be diagnosed externalizing behavior problems in preschool-aged children and their underestimated impact on parenting stress. For parents of children with clinically diagnosed conditions, such as parents of children with SHCN [48] or ASD [49], considerable clinical efforts have been made to provide parenting stress management strategies. Given our findings indicating an increased risk of elevated parenting stress associated with the child externalizing behavior problems, even among parents of children without clinically diagnosed conditions, these efforts should be extended to broader parent populations. 

A few limitations of this study should be acknowledged. First, we did not consider the severity of clinical conditions, which might have confounded the findings among children with clinical conditions. Second, the externalizing behavior measure used may not have fully captured comprehensive externalizing behaviors, which could have caused non-differential misclassification. Third, our findings could be confounded by parents’ stress coping strategies, as it is known that parents who adopt positive coping strategies are less likely to develop stress [50]. We were unable to account for the effects of parents’ coping strategies because coping strategy data were unavailable. Lastly, MEDB problem diagnosis was based on parent recollection and was not independently verified, which may have caused recall bias. Because parents with lower health literacy were likely to have larger recall bias, differential misclassification may have occurred. In addition, because some MEDB problems, such as a learning disability, are typically not diagnosed for preschool-aged children in US pediatric practices, child participants might have had undiagnosed MEDB problems, which could have led to misclassifications of MEDB problems.

## 7. Conclusions

This study confirms that elevated parenting stress is common among parents of preschool-aged children with SHCN and with clinically diagnosed MEDB problems. This study also suggests that externalizing behavior problems among preschool-aged children are associated with elevated parenting stress, regardless of the presence of children’s clinical conditions. The finding of the positive association between child externalizing behavior problems and elevated parenting stress even among families of children without clinical conditions calls for greater attention to subclinical or yet to be diagnosed externalizing behavior problems among the general preschool-aged child population and their underestimated impact on parenting stress.

## Figures and Tables

**Table 1:** Comparisons of participant characteristics by parenting stress status in US families of children aged 3–5 years. 2018–2019 National Survey of Child Health.

	Elevated Parenting Stress	No Elevated Parenting Stress	*p*-Value
	Unweighted n (Weighted %)	Unweighted n (Weighted %)	
All	403 (5.1)	8051 (94.9)	
Child age			0.72
3 years	135 (4.8)	2737 (95.2)	
4 years	143 (5.6)	2641 (94.4)	
5 years	125 (4.9)	2673 (95.1)	
Child sex			<0.01
Male	277 (6.6)	4117 (93.4)	
Female	126 (3.6)	3934 (96.4)	
Child race/ethnicity			0.28
Non-Hispanic white	261 (4.7)	5671 (95.3)	
Hispanic	50 (4.2)	924 (95.8)	
Non-Hispanic black	26 (7.5)	377 (92.5)	
Asian	22 (5.7)	409 (94.3)	
Other	44 (6.4)	670 (93.6)	
Family structure ^a^			0.05
Two parents, currently married	288 (4.3)	6330 (95.7)	
Two parents, not currently married	38 (6.2)	656 (97.8)	
Single parent	71 (7.4)	1046 (92.6)	
Family income			<0.01
FPL < 100 (below poverty)	58 (9.9)	661 (90.1)	
100 ≤ FPL < 200	62 (3.7)	1347 (96.3)	
200 ≤ FPL < 400	141 (4.4)	29.1 (95.6)	
FPL ≥ 400	142 (4.5)	3142 (95.5)	
Medical insurance for the child throughout the past year ^b^			0.12
Yes	375 (4.9)	7584 (95.1)	
No	26 (7.7)	408 (92.3)	
Child medical insurance type ^c^			0.07
Private	246 (4.2)	5981 (95.8)	
Public or public plus private	136 (6.1)	1709 (93.9)	
Uninsured	16 (7.7)	282 (92.3)	
Parent age			0.72
20–31 years	100 (5.6)	1790 (94.4)	
32–40 years	219 (4.8)	4654 (95.2)	
≥41 years	84 (5.2)	1607 (94.8)	
Parent sex			0.23
Male	118 (4.4)	2490 (95.6)	
Female	285 (5.4)	5534 (94.6)	
Parent education			0.12
≤High school graduation	183 (5.7)	3190 (94.3)	
<High school graduation	220 (4.4)	4861 (95.6)	
Parent birthplace ^d^			0.95
Born in US	344 (5.1)	6925 (94.9)	
Born outside US	58 (5.0)	1108 (95.0)	
Emotional support for parent			0.13
Yes	303 (4.7)	6918 (95.3)	
No	98 (6.3)	1113 (93.7)	

^a^ Missing n = 25; ^b^ missing n = 61; ^c^ missing n = 84; ^d^ missing n = 19; FPL, federal poverty level.

**Table 2:** Comparisons of elevated parenting stress by child’s SHCN, MEDB problems, and externalizing behavior problems in US families of children aged 3–5 years. 2018–2019 National Survey of Child Health.

	All	Elevated Parenting Stress	No Elevated Parenting Stress	*p*-Value
	Unweighted n	Unweighted n (Weighted %)	Unweighted n (Weighted %)	
SHCN				<0.01
Yes	1227	218 (20.8)	1009 (79.2)	
No	7227	185 (2.7)	7042 (97.3)	
MEDB problems				
Yes	1020	232 (24.8)	788 (75.2)	
No	7434	171 (2.4)	7263 (97.6)	
Externalizing behavior problems ^a^				<0.01
Yes	2509	332 (12.5)	2177 (87.5)	
No	5735	56 (1.4)	5679 (98.6)	
(EBQ1) Calming down quickly when excited or all wound up ^a^				<0.01
Never or sometimes	972	197 (19.8)	775 (80.2)	
Half the time or more	7303	191 (3.0)	7112 (97.0)	
(EBQ2) Being able to sit still ^b^				<0.01
Never or sometimes	838	184 (21.8)	654 (78.2)	
Half the time or more	7437	204 (2.9)	7233 (97.1)	
(EBQ3) Being easily distracted ^c^				<0.01
Always or most of the time	975	204 (18.4)	771 (81.6)	
Half the time or less	7295	185 (3.0)	7110 (97.0)	
(EBQ4) Losing control of his/her temper when things do not go his or her way ^d^				<0.01
Always or most of the time	936	180 (17.2)	756 (82.8)	
Half the time or less	7341	210 (3.3)	7131 (96.7)	
(EBQ5) Angry when going from one activity to another ^e^				<0.01
Always or most of the time	274	82 (21.9)	192 (78.1)	
Half the time or less	8006	309 (4.2)	7697 (95.8)	

^a^ Missing n = 210; ^b^ missing n = 179; ^c^ missing n = 184; ^d^ missing n = 177; ^e^ missing n = 174; EBQ, externalizing behavior question; MEDB, mental, emotional, developmental, or behavioral; SHCN, special health care needs.

**Table 3:** Adjusted odds ratios of elevated parenting stress in US families of children aged 3–5 years. 2018–2019 National Survey of Child Health.

	Model 1 (n = 8432)	Model 2 (n = 8244)	Model 3 (n = 1602)	Model 4 (n = 6642)
	AOR (95% CI)	AOR (95% CI)	AOR (95% CI)	AOR (95% CI)
Child sex				
Boy	1.3 (0.8, 2.0)	1.1 (0.7, 1.8)	1.2 (0.6, 2.4)	1.1 (0.6, 2.0)
Girl	Reference	Reference	Reference	Reference
Family income				
FPL < 100 (below poverty)	1.7 (1.0, 3.0)	1.3 (0.7, 2.4)	0.9 (0.5, 1.9)	2.2 (1.0, 5.1)
FPL ≥ 100	Reference	Reference	Reference	Reference
SHCN				
Yes	3.1 (2.0, 5.0)	2.3 (1.3, 3.9)	2.8 (1.3, 6.1)	NA
No	Reference	Reference	Reference	NA
MEDB problems				
Yes	6.7 (4.0, 11.0)	4.8 (2.5, 9.1)	6.7 (2.8, 16.0)	NA
No	Reference	Reference	Reference	NA
Externalizing behavior problems				
Yes	NA	5.4 (3.1, 9.4)	4.2 (1.9, 9.3)	6.4 (3.3, 12.7)
No	NA	Reference	Reference	Reference

Model 1: Elevated parenting stress = child sex + family income + SHCN + MEDB problems. Model 2: Elevated parenting stress = child sex + family income + SHCN + MEDB problems + externalizing behavior problems. Model 3 (subsample analysis for participants with clinical conditions): Elevated parenting stress = child sex + family income + SHCN + MEDB problems + externalizing behavior problems. Model 4 (subsample analysis for participants without clinical conditions): Elevated parenting stress = child sex + family income + externalizing behavior problems. AOR, adjusted odds ratio; CI, confidence interval; FPL, federal poverty level; MEDB, mental, emotional, developmental, or behavioral; NA, not applicable; SHCN, special health care needs.

## Data Availability

The National Survey of Child Health data are publicly available at https://www.childhealthdata.org/learn-about-the-nsch/NSCH (accessed on 8 February 2022).

## Supplementary Materials

The following supporting information can be downloaded at: https://www.mdpi.com/article/10.3390/children9020241/s1, Table S1: Prevalence of mental, emotional, developmental, and behavioral (MEDB) Problems in children aged 3–5 years. 2018–2019 National Survey of Child Health; Table S2: The prevalence of elevated parenting stress according to the number of externalizing behavior problems in families of children aged 3–5 years. 2018–2019 National Survey of Child Health; Table S3: Adjusted odds ratios of elevated parenting stress in mothers and fathers of children aged 3–5 years. 2018–2019 National Survey of Child Health.
