# Supplementary material for: The Associations of Child’s Clinical Conditions and Behavioral Problems with Parenting Stress among Families of Preschool-Aged Children: 2018–2019 National Survey of Child Health
Source: Children (Basel). 2022 Feb 11;9(2):241. doi: 10.3390/children9020241 (PMC8869988; doi:10.3390/children9020241)
Supplement: Supplementary file 1 [file children-09-00241-s001.zip › children-1557715-supplementary.pdf]

Supplementary Data

Supplementary Table S1. Prevalence of mental, emotional, developmental, and behavioral (MEDB) Problems in children aged 3-5 years. 2018-2019 National Survey of Child Health.

Conditions	Prevalence (%)
Tourette Syndrome	0.03
Anxiety problems	1.85
Depression	0.14
Behavioral and conduct problem	3.74
Developmental delay	5.49
Intellectual disability	0.51
Speech or other language disorder	8.91
Learning disability	2.10
ASD	1.91
ADD/ADHD	1.76
CSHCN screener-based mental, emotional, and behavioral problems	5.13

ASD, Autism Spectrum Disorder; ADD/ADHD, Attention Deficit Disorder or Attention-Deficit/Hyperactivity Disorder; CSHCN, children with special health care needs.

Supplementary Table S2. The prevalence of elevated parenting stress according to the number of externalizing behavior problems in families of children aged 3-5 years. 2018-2019 National Survey of Child Health.

	All	Elevated parenting stress	No parenting aggravation	p-value
	Unweighted n	Unweighted n (weighted %)	Unweighted n (weighted %)	
The number of externalizing behavior problems				<0.01
0	5735	56 (1.4)	5679 (98.6)	
1	1551	77 (5.1)	1474 (94.9)	
2	579	103 (16.0)	476 (84.0)	
3	215	69 (25.6)	146 (74.4)	
4	104	51 (38.2)	53 (61.8)	
5	39	30 (69.6)	9 (30.4)	

Supplementary Table S3. Adjusted odds ratios of elevated parenting stress in mothers and fathers of children aged 3-5 years. 2018-2019 National Survey of Child Health.

	Mother respondents (n=5,685)	Father respondents (n=2,551)
	AOR (95% CI)	AOR (95% CI)
Child sex		
Male	1.1 (0.6, 2.0)	1.3 (0.7, 2.5)
Female	Reference	Reference
Family income		
FPL<100 (below poverty)	1.2 (0.6, 2.4)	2.3 (0.9, 5.6)
FPL≥100	Reference	Reference
SHCN		
Yes	2.3 (1.2, 4.3)	2.3 (1.1, 4.7)
No	Reference	Reference
MEDB problems		
Yes	4.5 (2.0, 9.8)	7.0 (3.4, 14.3)
No	Reference	Reference
Externalizing behavior problems		
Yes	5.2 (2.6, 10.6)	5.8 (2.7, 12.5)
No	Reference	Reference

AOR, adjusted odds ratio; CI, confidence interval; FPL, federal poverty level; MEDB, mental, emotional, developmental, or behavioral problem; SHCN, special health care needs.
